# Clinical aspects of mastication myalgia—an overview

**DOI:** 10.3389/fpain.2023.1306475

**Published:** 2024-01-09

**Authors:** Golnaz Barjandi, Johanna Svedenlöf, Hajer Jasim, Malin Collin, Britt Hedenberg-Magnusson, Nikolaos Christidis, Malin Ernberg

**Affiliations:** ^1^ Department of Dental Medicine, Karolinska Institutet, and the Scandinavian Center for Orofacial Neuroscience (SCON), Huddinge, Sweden; ^2^ Department of Orofacial Pain and Jaw Function, Eastman Institute, Stockholm, Sweden

**Keywords:** diagnosis, epidemiology, etiology, masticatory muscle, myalgia, treatment

## Abstract

Mastication myalgia is the most common cause of non-odontogenic pain in the orofacial region and is often associated with a reduced quality of life. The purpose of this review is to provide an overview of the clinical aspects of myalgia based on available research. The review includes epidemiological, diagnostic, and etiological aspects. In addition, the potential risk factors related to the transition from acute to chronic myalgia are explored and treatment strategies are presented for its management. As a result, this review may increase clinical knowledge about mastication myalgia and clarify strategies regarding prevention, diagnostics, and management to improve prognosis and reduce patient suffering.

## 1 Introduction

Pain is a complex and multi-faceted phenomenon shaped by biological and psychosocial factors (1). While acute pain (short term pain) serves as a protective mechanism crucial for our survival, chronic pain (pain lasting more than 3 months) lacks a clear biological purpose (2).

Chronic pain is considered a significant public health problem globally and approximately one in five people in USA and Europe are estimated to have chronic pain (3, 4). It is more common in women compared to men, implying potential gender-related nuances in its development and persistence (3, 4).

Temporomandibular disorders (TMDs) are the most common type of chronic pains in the orofacial region and include pathological conditions affecting the masticatory muscles, temporomandibular joints (TMJs) and their associated structures. Among these, pain in masticatory muscles i.e., mastication myalgia (M-TMD) is more frequently encountered compared to TMJ disorders. M-TMD is characterized by local or regional pain in the masticatory muscles, increased tenderness, chewing difficulties, and restricted mouth opening (5). It is a debilitating condition often accompanied by a reduced quality of life for the patients. Similar to other pain conditions, M-TMD is more common in women compared to men and often co-occurs with other chronic painful and non-painful conditions of neurological, psychological, and gastrointestinal origin (6–8).

Although some patients, often those with localized M-TMD, have complete remission, the persistence of pain in the masticatory muscles is common (9–11). These cases frequently present a clinical challenge due to their complexity and the limited understanding of their origins and development pathways. Managing these patients proves complex due to the restricted range of treatment options, further aggravated by the occurrence of other comorbid health conditions, for example other pain conditions, depression, anxiety, stress, and sleep disruptions. Consequently, persistent pain and M-TMD is a frequent cause of sick leave, imposing substantial financial strains on the society, in the form of healthcare usage as well as reduced work and productivity (12).

This review aims to provide an up-to-date clinical overview of M-TMD including its epidemiology, etiology, and diagnosis as well as treatment approaches aimed at mitigating its incidence. Increasing our knowledge of M-TMD may facilitate early detection, effective management, and could potentially lead to reduced chronicity.

## 2 Prevalence and incidence

The prevalence of M-TMD is difficult to estimate accurately, as it varies widely depending on differences in study cohorts, diagnostic criteria, and examination methods (13, 14). However, research suggests that it is a relatively common condition worldwide and the lifetime prevalence is between 3% and 15% in the general population. Nevertheless, the prevalence of M-TMD can be higher in specific subgroups with conditions, such as fibromyalgia, chronic fatigue, or whiplash syndrome (13, 15, 16).

Several studies have investigated the prevalence and incidence of orofacial pain in individuals between 10 and 80 years old (17–20). The prevalence varies considerably depending on how the question was posed. Studies that ask for TMD symptoms generally show higher prevalence, while studies asking for more frequent orofacial pain in general show lower prevalence. For example, a large study in Sweden including 137,000 individuals reported an overall prevalence of frequent orofacial pain (at least once per week) of almost 5% (18). Fewer studies have specifically investigated the prevalence of M-TMD. A meta-analysis of 21 studies including a total of 3,463 patients with TMD found an overall prevalence of M-TMD of 45.3%, but a prevalence of 9.7% in studies conducted in the general population (2,491 subjects) (17). A study from Brazil where 1,643 subjects were randomly selected and examined according to the RDC/TMD criteria revealed that 29.5% fulfilled the criteria for M-TMD (19). The prevalence of signs and symptoms of M-TMD in children and adolescents is even more variable due to the absence of a validated diagnostic criteria for this specific population.

Numerous studies consistently demonstrate a higher prevalence of M-TMD in females compared to males (13, 17, 18) with women being two to three times more likely to suffer from M-TMD than men. However, the gender differences are greater in samples drawn from tertiary clinics than from the population and one reason to this may be differences in health care seeking (21). There are also signs of increasing prevalence of orofacial pain over time and more so in women, indicating an increasing gender difference for orofacial pain including M-TMD (22).

M-TMD can occur at any age but is often reported to occur more frequently in young to middle-aged adults. The Orofacial Pain: Prospective Evaluation and Risk Assessment (OPPERA) study, a comprehensive cohort study conducted in the United States, provided estimates regarding the development of painful TMD in adults aged 18–44 years (23). The study revealed that approximately 4% of healthy pain-free adults within this age range develop clinically confirmed first-onset painful TMD each year. Furthermore, the annual incidence of painful TMD was observed to increase with age, with rates of 2.5% for those aged 18–25 years, 3.7% for those aged 25–34 years, and 4.5% for those aged 35–44 years (23). While M-TMD can also affect elderly individuals, its frequency appears to decrease in this population (20). In such cases, myalgia is particularly associated with age-related changes in the musculoskeletal system or may be secondary to osteoarthritis.

## 3 Etiology

There is not one specific cause of TMD, and studies have shown TMD to have a biopsychosocial and multifactorial background with several risk factors that increase the susceptibility of developing TMD (16, 24–26). These factors can be biological, behavioral, psychosocial, but also previous chronic pain conditions and trauma are important risk factors. It has been suggested that one of the most important factors to develop TMD is other somatic symptoms (27). For instance, patients with fibromyalgia have a higher prevalence of TMD symptoms (28). Furthermore, having another chronic pain condition increases the risk of developing a new chronic pain disorder (21).

In addition to somatic symptoms, increased levels of anxiety, sleep difficulties and depression are often found in TMD patients making biopsychosocial aspects strong predictor for developingpainful TMDs (29). It has been hypothesized that pain amplification may be important in the etiology since it seems to occur more often in patients TMD-M. The pain amplification may be caused by enhanced pain facilitation and deficient pain inhibition. This is supported by increased mechanical sensitivity (lower pain thresholds), greater temporal summation, and impaired conditioned pain modulation in TMD compared to pain-free controls (21, 30). Biological sex is among identified risk factors for TMD-M, which is not surprising given the higher prevalence of orofacial pain in women than men in the general population (18, 22). However, other studies have reported only marginally increased risk for TMD in women (15, 31) and suggested that this may be due to prevalence-incidence bias (15). However, other factors such as sex hormones, psychosocial factors and gender role expectations may also explain these differences. Also, prior trauma, both macro- and microtrauma, such as prolonged mouth opening, intubation, and parafunctional activities have been suggested as a denominator of TMD etiology (24, 26). The OPPERA study found that parafunctional activity, reported by the patient, was a predictor for TMD (15, 32).

## 4 The transition from acute to chronic TMD pain

In TMD, the transition to chronic pain and the risk factors associated with the transition are important to understand for determination of a personalized treatment strategy.

A few studies have investigated differences between patients with acute and chronic TMD pain. Sabsoob and coworkers in a critical review including seven studies found no significant difference regarding age, race, gender and socioeconomical status between the groups (33). However, one of the included studies reported that chronic TMD pain was more prevalent in females than in males (34). Psychological factors such as depression, stress and somatization were more prevalent in chronic TMD pain patients, whereas anxiety showed conflicting results (33). The study by Cao et al. (34) also reported that patients with chronic TMD pain more often reported impaired sleep quality than patients with acute TMD pain. This indicates that poor sleep may be important for the transition from acute to chronic TMD pain. Further, chronic TMD pain patients more often had a diagnosis of TMD-M than acute TMD patients, while arthralgia was more common in acute TMD patients (33). Meloto et al. (2019) found that the vertical mobility of the mandible in combination with experience of a pain-sensation that was altered by mandibular movements was correlated with persistent TMD, indicating that hyperalgesia could be a predictor (35). The OPPERA study in a cluster analysis found three clusters in their cohort, an adaptive, a pain-sensitive with increased sensitivity to experimental pain, and a global symptoms cluster with increased sensitivity and greater psychological distress. The chronic TMD cases were more likely to belong to the pain-sensitive and global symptoms cluster and they showed greater pain intensity, jaw functional limitation, and more comorbid pain conditions than the cases in the adaptive cluster (36).

A diagnosis of TMD-M was found important for the transition from acute to chronic TMD pain. This risk appeared to be greater in women and was not confounded by pain intensity, pain disability, or non-specific symptoms (33). Another risk factor identified in the critical review (33) was pain intensity at baseline, which also has been reported previously in other pain conditions (37, 38). Also, patients with high grade of disability at baseline had an increased risk of developing chronic TMD, even if this risk did not reach significance (33). Finally, another study found that chronic neck pain was more prevalent in patients with chronic TMD pain and disability than acute TMD pain, suggesting that chronic disability might be involved in the transition from acute to chronic TMD pain (39). However, the factors involved in the transition from acute to chronic pain are almost unknown and much more research is needed to understand this in order to be able to prevent the transition.

## 5 Classification

Ideally diagnostics should be based on objective measures with high sensitivity and specificity for the disease in question. Objective measures require that the underlying mechanisms of the disease are known, for example through specific biomarkers. Biomarkers have been defined as “…characteristics that are objectively measured and evaluated as indicators of normal biological processes, pathogenic processes, or responses to an intervention” (40). This means that the measurement of blood pressure for diagnostics of hypertension or blood glucose level for diabetes can be classified as biomarkers.

For chronic pain, such as M-TMD, there is no single biomarker that can be used for diagnosis. Instead, we must rely on symptoms and signs specific for the disorder. Pain is the most prominent symptom and can be classified in many ways. For example, according to its anatomical location, such as headache or orofacial pain. As mentioned above, pain can also be classified as acute or chronic. In contrast to acute pain, which is regarded a symptom of a disease, chronic pain is defined as a disease in itself (41). Another way of classifying pain is by the supposed mechanism, for example nociceptive, neuropathic, and nociplastic pain. The term nociplastic pain was introduced in 2016 and describes a “pain that arises from altered nociception despite no clear evidence of actual or threatened tissue damage causing the activation of peripheral nociceptors or evidence for disease or lesion of the somatosensory system causing the pain” (42). In the suggested clinical criteria for nociplastic pain the presence of comorbidities (e.g., sleep disturbance and fatigue) and sensory disturbances in the region of pain (e.g., mechanical, or cold allodynia) are important for clinical diagnostics (43). These are common features of TMD patients. Therefore, at least a subgroup of patients with TMD could probably be classified as having nociplastic pain, while others may have nociceptive pain. It is also possible to have a combination of nociceptive and nociplastic pain and ongoing nociceptive pain has been suggested to be a risk factor for developing nociplastic pain (43, 44).

To diagnose TMD several classifications exist (Table 1). Many of these include a set of criteria that are not validated, e.g., the 2008 classification of orofacial pain by the American Academy of Orofacial Pain (AAOP) (45) that was intended to be used “as a road map” for clinical purposes. The Research Diagnostic Criteria for TMD (RDC/TMD) (46) introduced a paradigm shift as it considered the biopsychosocial model, and, in addition to the clinical diagnoses (axis I), included psychosocial assessment (axis II). The axis I includes validated examination methods. Thus, it was the first classification that was at least partly validated. Later validation, however, found that the sensitivity and specificity for the diagnoses were low (47, 48); only for myofascial pain and myofascial pain with limited opening combined, the validity reached the pre-set criteria. To try to overcome this, and to make the classification clinically more useful, the algorithms were revised and updated diagnoses were later presented in the Diagnostic Criteria for TMD (DC/TMD) (5). This classification includes validated criteria for the most common TMD diagnoses. The DC/TMD reached excellent sensitivity and specificity for M-TMD and the other pain diagnoses, but for the intracapsular TMJ disorders the preset limits for sensitivity and specificity were still not met. Nevertheless, it was suggested to be used in both research and clinical settings and is now the most used classification at specialist clinics world-wide with translations into many languages.

**Table 1:** Classifications that include M-TMD diagnoses.

Classification	Abbr.	Publ. yr	Validated criteria	Diagnoses
Research Diagnostic Criteria for TMD	RDC/TMD	1992	Yes, partly	Myofascial pain
				Myofascial pain with limited opening
The IASP Classification of Pain	IASP	2011	No	Temporomandibular Pain and Dysfunction Syndrome
Diagnostic Criteria for TMD	DC/TMD	2014	Yes	Myalgia Local myalgia Myofascial pain Myofascial pain with referral
Expanded Taxonomy		2014	Yes^a^	Myalgia Local myalgia Myofascial pain Myofascial pain with referral
				Myositis
				Tendonitis
				Spasm
American Academy of Orofacial Pain	AAOP	2018 (6th ed)	Yes, partly^a^	Myalgia Local myalgia Myofascial pain with spreading Myofascial pain with referral
				Tendonitis
				Myositis
				Spasm
The International Classification of Headache Disorders	ICHD	2018	No	Temporomandibular disorders
International Classification of Diseases	ICD-11	2018	No	Chronic primary headache or orofacial pain
				Chronic secondary headache or orofacial pain
International Classification of Orofacial Pain	ICOP	2020	No^b^	Myofascial orofacial pain
				Primary myofascial orofacial pain *Acute primary myofascial orofacial pain* *Chronic primary myofascial orofacial pain*
				Secondary myofascial orofacial pain *Myofascial orofacial pain attributed to tendonitis* *Myofascial orofacial pain attributed to myositis* *Myofascial orofacial pain attributed to muscle spasm*

^a^ DC/TMD criteria for myalgia and subdiagnoses.

^b^ DC/TMD criteria incorporated in diagnoses.

At the same time as the development of the DC/TMD an Expanded Taxonomy of the DC/TMD was developed and published (49). This is a broader classification of orofacial pain based on the AAOP classification for orofacial pain with expert-based criteria also for more rare conditions that awaits further research for validation. However, for M-TMD (and other DC/TMD diagnoses), the DC/TMD diagnoses and criteria were adopted (5). Also, the AAOP has adopted the DC/TMD in the 2018 edition (50) (Table 1).

Shortly after the work with DC/TMD, the International Association for the Study of Pain (IASP) began work to include pain diagnoses in the new version of the International Classification of Diseases (ICD-11) (51). In this, orofacial pain is included under the code MG30.03 (Chronic primary headache or orofacial pain) and MG30.6 (Chronic secondary headache or orofacial pain), but no specific TMD diagnoses are included.

Another classification is the International Classification of Headache Disorders (ICHD-3) which includes criteria for Headache attributed to TMD (52). However, it is stated that since diagnosis of TMD can be difficult, “the diagnostic criteria evolved by the International RDC/TMD Consortium Network and Orofacial Pain Special Interest Group is recommended”, i.e., the DC/TMD classification (5).

The most recent classification for orofacial pain is the International Classification of Orofacial Pain (53). This includes myofascial orofacial pain. The classification follows the structure of the ICHD criteria for tension-type headache where the pain is classified according to its frequency (infrequent, frequent, and highly frequent) but have included the validated DC/TMD criteria for diagnosis.

The DC/TMD has now been used for almost ten years and recently the DC/TMD for children and adolescents was published (54). This is the first classification of TMDs in children. It was developed after a Delphi process and includes the DC/TMD axis I. However, it needs to be validated in this patient group. The diagnostics according to DC/TMD require the use of specified commands and examination procedures, why it has shown difficult to implement it in general dentistry. A simplified version for general dentistry, which includes only the examinations of importance for pain diagnoses is under development (personal communication). This lacks the verbal commands as a study showed that excluding the commands did not impair the diagnostic reliability (55). The hope is that these new criteria will be used by general dentists. In Sweden, these criteria are now taught at all dental schools.

To screen for TMDs there are also a few instruments available. The TMD pain screener is a validated instrument that includes three (short version) or six (long version) questions about the presence of orofacial pain in general, pain or stiffness at awakening, and activities that may influence pain during the last 30 days (56). A score of 2 out of 4 for the short version and 3 out 7 for the long version are cut-off values. Another validated screener is the 3Q/TMD (57). This also includes three questions, two concerning the presence of orofacial pain in general and at jaw opening once per week or more often, and one question about jaw catching/locking, once per week or more often. A positive answer to any of these questions imply the need for further interview and examination of the jaw system. The use of a short screening questionnaire together with a shorter DC/TMD examination is expected to facilitate diagnostics for practicing general dentists.

## 6 DC/TMD myalgia diagnostics

For M-TMD, the DC/TMD lists four diagnoses, of which two are validated. The sensitivity and specificity for myalgia are 0.90 and 0.99, respectively, and for myofascial pain with referral, 0.86 and 0.98, respectively (5). With the publication, two additional diagnoses were included without being validated, local myalgia and myofascial pain. The structure of the DC/TMD is built on both subjective and clinical criteria. For the pain diagnoses these are very similar for all diagnoses, which facilitates learning.

What was new in the DC/TMD compared to the RDC/TMD (46) is that the diagnostic algorithm for myalgia is based on pain in the temporalis or masseter muscle only, no other jaw muscles are included. The clinical DC/TMD examination includes confirmation of pain and headache location; mandibular movement capacity and pain on movements, palpation for TMJ sounds, and palpatory pain of the TMJ, masseter, and temporalis muscle (5). With the diagnostic criteria structured and clearly specified examination procedures are included, for example verbal commands and specification of palpatory time and pressure.

The four DC/TMD myalgia diagnoses are based on subjective reports of pain in the jaw, temple, ear, or in front of the ear during the last 30 days, which is modified with jaw movement, function, or parafunction. During the clinical examination the examiner confirms that the pain area is located within the masseter or temporalis muscle, and the patient report if familiar pain in the masseter and temporalis muscle during jaw opening or palpation of these muscles is present. Depending on if muscle palpation elicits pain that only is experienced in the point that is being palpated, or spreads within or beyond the muscle (for example to the teeth) the sub-diagnoses local myalgia, myofascial pain, and myofascial pain with pain referral can be determined (5).

In DC/TMD, as well as in its predecessor the RDC/TMD psychosocial factors are assessed with the axis II (5, 46). This includes instruments for assessment of pain intensity and pain interference (Graded Chronic Pain Scale), oral parafunctions (Oral Behavior Checklist), Jaw function (Jaw Function Limitation Scale), distress (Patient Health Questionnaire-4 or -9), anxiety (General Anxiety Disorder-7), and physical symptoms (patient History Questionnaire-15).

## 7 Treatment approaches

It is important to remember that every individual experience pain uniquely and, therefore, treatment regimens need to be individually tailored for each patient (58). Although several treatment approaches have been shown to be successful in management of M-TMD (59), to our knowledge, no single treatment approach has been found superior to all other approaches. Thus, a comprehensive biopsychosocial treatment approach is recommended (60, 61).

According to a recent network meta-analysis (62), the most effective treatment approaches in M-TMD seem to be counseling, occlusal appliances, and jaw exercises, manual therapy or physiotherapy (Table 2). An important point of note is that all these treatments are minimally invasive and for the most part reversible. Other suggested treatment approaches for M-TMD include e.g., acupuncture and needling therapy (63), oral pharmacological treatments (64), behavioural therapies (65), and physical therapies such as for instance transcutaneous electrical nerve stimulation (TENS) (66)and thermal therapy (67).

**Table 2:** Ranking of TMD treatments according to best evidence.

Treatment	Level of evidence	SMD (95% CI)
Occlusal appliance	Moderate	−0.94 (−1.38; −0.51)
Manual	Low	−1.10 (−1.69; −0.51)
Counceling	Low	−0.95 (−1.52; −0.38)
Ozon	Very low	−1.11 (−1.95; −0.27)
Local anesthetics	Very low	−0.77 (−1.41; −1.12)
Botulinum toxin	Very low	−0.72 (−1.25; −1.19)
Dry needling	Very low	−0.70 (−0.27; −0.14)
Hypnosis	Very low	−0.67 (−1.59; 0.24)
Laser	Very low	−0.58; (−1.30; 1.13)
Muscle relaxants	Very low	±.48 (−1.09; 0.13)

SMD, Standard mean difference; CI, Confidence interval. Adapted after Al-Moraissi et al. (62).

Considering the wide range of available therapies, the everyday clinical decision-making can be a hassle and difficult. Therefore, clinical treatment guidelines can be helpful. In Sweden, for instance, the Swedish Academy of Temporomandibular Disorders, has developed a national treatment protocol for general dentists regarding TMD including M-TMD (68). The national treatment protocol for TMD is based on the Swedish National Guidelines in Dentistry, by The Swedish National Board of Health and Welfare (69), that in turn are based on current best evidence (science and proven experience).

A short overview of the most common treatment approaches will follow.

### 7.1 Information and counseling

When planning a treatment, it is of great importance to set realistic treatment goals and choose the most suitable treatment strategy for the individual patient. The purpose of the treatment should be to eliminate or reduce the effect of the underlying causes and not just alleviate the symptoms or consequences of these. Since M-TMD is multifactorial with both functional, structural, and psychological causal factors great emphasis should be placed on information and counseling (70).

According to science and proven experience, patients with M-TMD should be offered information and advice as a first step in their treatment (68, 69). Treatment should always begin by giving the patient a simple and clear explanation of the condition, the causal relationships that exist, and that M-TMDs are common, benign conditions, with a good prognosis. Information and counseling can be calming, which in turn can lead to a reduction in anxiety, helping the patient to better tolerate their pain. Information and advice that includes instructions on self-care should be provided together and in a sequence. After information about the condition and causal relationship, counseling should be offered regarding M-TMD followed by individualized advice on self-care (71). One has not to forget that stress management and sleep hygiene also can be included in counseling. This, since it is well known that disturbed sleep increases pain sensitivity (72, 73), and that incorporating physical activity such as forest walks has proven to relive stress and improve overall physical wellbeing (74).

Finally, it is common that patients are unaware of possible parafunctions, or when such parafunctions occur. Therefore, it is important to make the patients aware of their parafunctions and to justify why they need to reduce their parafunctions to motivate them (71). For example, reduction of chewing or clenching, nail-biting, cheek- or lip-biting can in many cases be enough to relieve patients' M-TMD pain.

### 7.2 Behavioral therapy

Orofacial behaviors encompass mastication, communication (verbally and by facial expressions), and nonfunctional behaviors, such as excessive overuse and parafunctions. The importance of addressing orofacial behaviors in the management of M-TMD has been recognized since the 1950s (75), with increasing evidence since then (75, 76). The management include techniques to create awareness regarding the patients' own body's functions and parafunctions. One commonly used technique is biofeedback. Usually, different kinds of devices are used to register muscle activity and when activity occurs this is signaled to the patients, thus encouraging them to observe or change their behavior. Although time consuming, biofeedback is shown to be effective in reducing masticatory muscle activity (77). However, there is no consensus regarding biofeedback and pain alleviation in M-TMD.

Visual feedback, on the other hand, is a simple and cost-effective method to change a parafunctional behavior. By first identifying situations and/or places where muscle tension or parafunction are induced, a visual reminder, i.e., a colorful sticker is placed there so the patients constantly become reminded and prompted to change their behavior. These situations or places can for instance be working on the computer, driving a car, or using a phone or a tablet. Recently digital applications that automatically send reminder to the patient have been developed.

### 7.3 Occlusal therapy

Occlusal appliances are commonly used in the treatment of painful TMDs, with the stabilization appliance being the one most used (78). A recent meta-analysis showed that there is moderate evidence that the stabilization appliance reduces M-TMD pain (79), and it has recently been shown as being among the most effective treatments of M-TMD (62). Further, its treatment effect is better than placebo, which is not the case with other types of occlusal appliances (80).

As the mechanisms of action still are unclear, some possible mechanisms that could explain the beneficial effects of stabilization appliances have been proposed. There could be a change in the reflective pattern of the chewing muscles, an increase in awareness of parafunctional activity in the patients, a decrease of the load of the chewing muscles, and last but never to be forgotten, the placebo effect (81–83).

Though occlusal adjustment is an irreversible treatment and there is no evidence that it is effective in treating M-TMD (84), it is still used by dentists, especially in general practice. Since the treatment is doing more harm than good it should not be used to treat M-TMD (85).

### 7.4 Physiotherapy and self-exercise

Individualized self-exercises for the jaw has been shown to relieve pain both in localized/regional M-TMD and M-TMD associated with generalized pain (86, 87). Further, combining jaw exercises with counselling and relaxation is even more effective (87–89). Self-exercise modalities can be categorized as mobilization exercise, muscle strengthening exercise (resistance training), coordination exercise, and postural exercise. Mobilization and coordination exercises aim to improve muscle flexibility and normalize imbalanced muscle activity by changing patterns of movement and thus relieve muscle pain. Isometric resistant training on the other hand, may relieve muscle pain through an inhibitory effect on muscle activity via Golgi tendons (90). As with most treatments, the outcome of self-exercises regimes heavily relies on the degree of compliance and should therefore be structured, supervised, and evaluated to be effective (86).

### 7.5 Pharmacological therapy

#### 7.5.1 Per oral

Analgesics are considered a good complement to other treatment modalities for M-TMD. Non-opioid analgesic drugs, which are relevant in this context, is a heterogeneous group mainly consisting of paracetamol and anti-inflammatory drugs. These are recommended for patients with mild to moderate M-TMD pain (91–93).

The analgesic mechanism of action for paracetamol is not fully known. What is known is that paracetamol inhibits the prostaglandin synthesis in the central nervous system, and that it acts via the cannabinoid system (94). Since paracetamol does not act on the cyclooxygenase enzyme (COX) it has no anti-inflammatory effect. Nonsteroidal anti-inflammatory drugs (NSAIDs), on the other hand, inhibit both the enzymes COX-1 and COX-2. The different NSAIDs have varying selectivity for the COX enzymes. By inhibiting the COX enzymes, prostaglandin synthesis is inhibited which in turn reduces inflammation and pain signalling (95). Even though naproxen and ibuprofen (COX-1) are considered the safest NSAIDs to use when it comes to the cardiovascular system, they have been shown to have adverse effect on the gastrointestinal tract causing ulcers and gastric bleeding (92, 96). On the other hand, the COX-2 inhibitor celecoxib has shown to have a small risk for gastrointestinal bleeding, but a high risk for cardiovascular disease (92). So, if the patient is at risk for cardiovascular disease the first-line NSAID choice is naproxen or ibuprofen. If the patient, on the other hand, is at risk for gastrointestinal bleeding and at no risk for cardiovascular disease the first-line NSAID choice is celecoxib.

Another proposed approach is the use of muscle relaxants. Even though they have been used for years to treat M-TMD pain there is very little evidence of an effect, and it seems to be minimal (62, 97). Their usefulness is also limited due to the reported side effect in the form of, for example, drowsiness, dizziness, weakness, and ataxia (98, 99). The mechanism of action of muscle relaxants is that they reduce muscle tension. Therefore, they can sometimes be indicated for reduction of muscle spasms, muscle pain, and headaches. However, a recent network meta-analysis reported that the muscle-relaxant substance cyclobenzaprine could have a short-term pain reducing effect in M-TMD (97). Muscle relaxants must be used with caution since they may induce a significant degree of sedation. Further, these kind of medications are contraindicated for patients with hyperthyroidism and/or congestive heart failure, or patients using monoamine oxidase inhibitors and tramadol (92).

For chronic pain, antidepressants, such as amitriptyline are often recommended and proved to be effective (100). However, a recent network meta-analysis showed that duloxetine was superior to other antidepressants for chronic pain treatment having moderate to high certainty evidence (101). The antidepressants exert their analgesic effect by blocking the re-uptake of serotonin, and/or noradrenalin in doses lower than their antidepressant effect (100). Studies investigating antidepressants in the treatment of M-TMD are scarce. However, a systematic review concluded that there is weak evidence that they are effective but that more studies with sound methodology are needed to demonstrate their effectiveness (102). There are also side-effects to consider with these kinds of drugs. For instance, tricyclic antidepressants are associated with dizziness, blurred vision, constipation, sedation, and dry mouth (103). As for muscle relaxants, tricyclic antidepressants should be avoided for patients using or patients using monoamine oxidase inhibitors since this combination of medications has, in worst case scenario, been shown to lead to lethal serotonin syndrome, which is a condition consisting of confusion, fever, ataxia, as well as severe hypertension (92, 104). Finally, tricyclic antidepressants should be used with caution among elderly and for patients with cardiac disease (92). Even the selective serotonin re-uptake inhibitors should be used with caution since they have been shown to cause dry mouth, nausea and vomiting, headaches, sexual dysfunction, and sweating (105).

#### 7.5.2 Local

Although an increasing number of patients with M-TMD are treated with botulinum toxin, the evidence for its pain reducing effect is still sparse and equivocal (106). Results from the most recent meta-analysis and two netwok meta-analyses indicate that botulinum toxin might have a pain reducing effect, at least in a long-term perspective (62, 107, 108). Though the evidence is very low, more recent RCTs with good methodology seem to show more favourable effects on M-TMD (109, 110). Botulinum toxin is considered safe with few serious side-effects (106). Nevertheless, headache is a known short-term side effect, that is induced as the toxin initially causes muscle spasm and then complete paralysis (111). Further, multiple (112–114), and even a single dose (109) of botulinum toxin causes muscle atrophy and decreased bone volume at the mandibular condyle and body. Because of the low evidence for an effect and high cost, botulinum toxin should be used restrictively.

Similar to botulinum toxin the evidence for a pain-reducing effect by local anaesthetics M-TMD is, very low both in a short- and a long-term perspective (62). Other agents, such as granisetron (a serotonin receptor type 3 antagonist) and platelet rich plasma are reported effective in M-TMD, but also here the evidence is very low due to the few studies done so far (62). The included studies in this review did not report any side-effects for local anaesthetics, granisetron or platelet rich plasma (62). Taken together, future studies on the effect of local pharmacological treatments in M-TMD are needed.

### 7.6 Needling therapy

Needling techniques include acupuncture and deep dry needling (115, 116). Though acupoints often coincide with myofascial trigger points, acupuncture and dry needling are different therapies (117). In acupuncture thin filiform and dry needles are inserted into defined acupoints in meridians and non-trigger points (115, 118), to induce a better flow of the energy (qi) (119). In deep dry needling, on the other hand, a thin monofilament needle is directly inserted in myofascial trigger points to inactivate them (116).

Even though several studies have shown that acupuncture and deep dry needling are effective, minimally invasive, cheap, and easy to learn treatment modalities for musculoskeletal pain conditions (120), these studies have been carried out in larger muscle-groups as well as in areas outside the orofacial region (121). When the pain reducing effect of acupuncture and deep dry needling was investigated and compared to active or inactive placebo for TMD treatment in a recent network meta-analysis no beneficial effects were found neither regarding pain reducing effect, nor mechanical sensitization (122). However, on an individual basis both acupuncture and deep dry needling can have a pain reducing effect in M-TMD.

### 7.7 Transcutaneous electrical nerve stimulation

TENS is an effective non-invasive method that promote analgesia and a decrease in EMG activity of masticatory muscles at rest (123). The mechanisms by which TENS exert its effects is believed to be by activation of “gate control” (high-frequency TENS, 50–120 HZ) or by inducing release of endogenous opioids (low-frequency TENS, 2 Hz). A recent systematic review including eight studies with painful TMDs found a significant and considerable pain reduction and improvement of mouth opening immediately after TENS treatment and that the pain intensity was still 40% lower than at baseline one month after completion of treatment (124). In M-TMD, and for treatment of the masseter and temporalis muscles high frequency TENS therapy is recommended (123). However, there are no consensus on an optimal TENS protocol for M-TMD; clinicians are advised to follow manufactures recommendations and regular follow-ups are essential with TENS therapy.

## 8 Future directions

Ongoing research is actively exploring various dimensions of M-TMD, offering prospects to improve diagnostic, management, and prognostic approaches.

In this regard, the field of genetics and proteomics emerges as a promising avenue. An evolving taxonomy would benefit from embracing a mechanistic classification that not only segregates pain disorders by M-TMD signs and symptoms, but also factors in the underlying mechanisms. This enhanced diagnostic framework has the potential to elucidate the diversity in prognosis and treatment response observed within existing diagnostic categories. For instance, identifying local and systemic biomarkers specific to M-TMD could enhance diagnostic precision and treatment strategies.

Moreover, future research in the heritability of M-TMD may also lead to personalized treatment modalities, grounded in individual genetic profiles. The complex mechanisms connecting genes, epigenetic factors, and masticatory muscle pain certainly need further research (125). Although there are conflicting results regarding the roles of genetic markers for M-TMD, studies indicate that certain gene polymorphisms may be associated with M-TMD. Therefore, “omics” research, i.e., studying genomic, transcriptomic, metablomic, and proteomic markers hold significant potential for future investigation into acute and chronic TMD development, which could facilitate early identification of individuals predisposed to M-TMD (126).

Advancements in brain imaging could also improve management of those with M-TMD. For instance, functional imaging techniques have previously been found distinctive patterns of functional and structural changes within the “pain matrix” (i.e., thalamus, the insular cortex, the primary and secondary somatosensory cortices, the anterior cingulate cortex, and the prefrontal cortex) in patients with chronic M-TMD (127). This emphasizes the potential of brain imaging to increase our knowledge of M-TMD.

Lastly, strategies targeting the transition from acute to chronic pain, lifestyle modifications, and early interventions could potentially mitigate the severity and occurrence of M-TMD. Building on the studies examining M-TMD, future research should delve deeper into stressful life factors, biopsychosocial aspects as well as genetics and pathophysiological changes of the diagnoses to get insights into the etiological factors driving the transition from acute to chronic M-TMD.

## 9 Conclusion

M-TMD is a complex condition that usually represents a clinical challenge, both in terms of diagnosis and treatment, but also due to lack of deeper knowledge about the etiology. Precise diagnosis is important and, the absence of an accurate diagnosis, may lead to treatment failure and perhaps even exacerbation of the condition. It is therefore important that healthcare professionals know how to recognize risk factors and provide accurate early diagnosis as well as appropriate management to prevent the acute M-TMD to become a chronic condition.

Moreover, this paper shed light on the various factors that contribute to the conditions, including muscular and psychological factors. The diagnostic process for M-TMD can be challenging and requires a thorough evaluation of the patient symptoms and medical history. Treatment options for M-TMD range from conservative measures to more invasive interventions. Therefore, it is important that healthcare providers understand and are familiar with the classifications of M-TMD and its treatments as well as the multifactorial etiology of each patient, to offer comprehensive, multifaceted treatment strategy for best prognosis of these patients.
